# Protocol for isolation of the brain vasculature from the mouse cortex for proteomics

**DOI:** 10.1016/j.xpro.2026.104729

**Published:** 2026-07-21

**Authors:** Yazi Huang, Veronica Clementel, Mingzi Zhang, Kate Martinez, Karen Martinez, Gavin Spillard, Carina Torres-Sepulveda, Kassandra Kisler, Marcelo P. Coba, Ruslan Rust

**Affiliations:** 1 Department of Physiology and Neuroscience, Keck School of Medicine, University of Southern California, Los Angeles, Los Angeles, CA 90033, USA; 2 Zilkha Neurogenetic Institute, Keck School of Medicine, University of Southern California, Los Angeles, Los Angeles, CA 90033, USA; 3 Department of Psychiatry and Behavioral Sciences, University of Southern California, Los Angeles, Los Angeles, CA 90033, USA

**Keywords:** Cell Biology, Cell isolation, Cell separation/fractionation, Genomics, Microscopy, Neuroscience, Protein Biochemistry, Proteomics, Mass Spectrometry

## Abstract

The neurovascular unit, composed of brain endothelial cells, pericytes, and astrocytes, regulates molecular exchange between blood and the central nervous system. Here, we present a protocol to isolate brain vessels from the mouse cortex using mechanical homogenization and dextran density-gradient centrifugation. We describe steps for cardiac perfusion, meningeal and white matter removal, homogenization, and separation of capillary-depleted parenchyma. We detail procedures for validating purity by immunofluorescence and western blot followed by sample preparation for liquid chromatography-tandem mass spectrometry (LC-MS/MS) proteomics.

For complete details on the use and execution of this protocol, please refer to Huang et al.^1^

## Highlights

• Steps for transcardial perfusion and isolation of mouse cortex for vessel work
• Mechanical homogenization and dextran density-gradient separation of brain vessels
• Instructions for purity assessment by immunofluorescence and western blot
• Guidance on sample preparation of brain vessels for LC-MS/MS proteomics

## Abstract (editor highlights)

Publisher’s note: Undertaking any experimental protocol requires adherence to local institutional guidelines for laboratory safety and ethics.

## Graphical abstract

**Figure:**
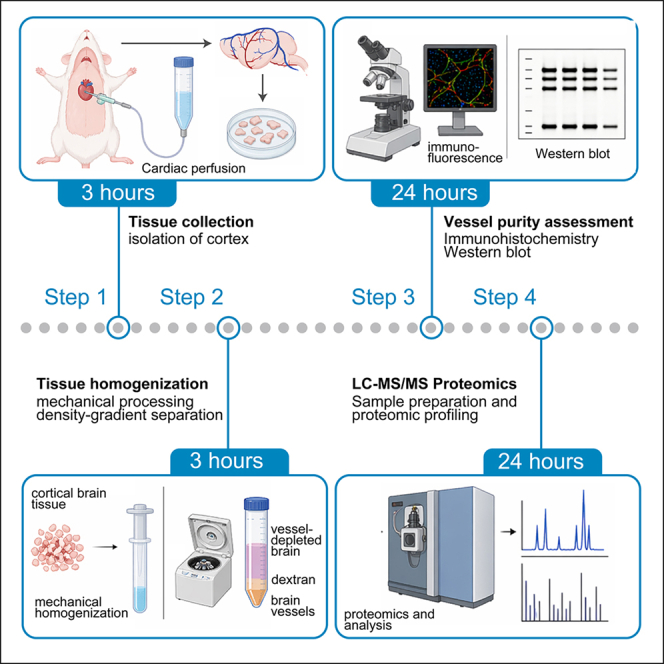

## Before you begin

This protocol describes the isolation of brain vessels from the mouse cortex and validation of yield and purity by immunofluorescence, western blot, and downstream sample preparation for LC-MS/MS proteomics. The workflow includes preparation of buffers and anesthesia, transcardial perfusion, meningeal and white matter removal, mechanical homogenization, dextran density-gradient centrifugation, cell strainer purification, cytospin-based immunofluorescence, and protein analysis by western blot. The protocol was developed for adult C57BL/6J mice but has also been used for mice carrying Notch3 point mutations (CADASIL models) as described in Huang et al.^1^

### Protocol timeline

1. Up to 4 weeks before experiment: Prepare buffer A and buffer B (store at 4°C, valid for 4 weeks).
2. Up to 3 months before experiment: Prepare 30% dextran solution (store at 4°C, valid for 3 months).
3. Up to 1 week before experiment: Prepare ketamine/xylazine working stock (store at 4°C, valid for 1 week).
4. Day 1 (approximately 3–4 h): Transcardial perfusion, brain collection, meninges and surface vessel removal, white matter removal, homogenization, dextran density-gradient centrifugation, and filtration. – Pause point: Brain vessel suspension can be held on ice for up to 2 h after filtration, or snap-frozen at −80°C for long-term storage.
5. Day 1–2: Downstream validation and analysis. – Immunofluorescence staining: 18 h (including 12–16 h primary antibody incubation at 4°C). – Western blot: 24 h (including 12–16 h primary antibody incubation at 4°C). – LC-MS/MS sample preparation: 2 h (plus shipment on dry ice if required).

### Innovation

Brain vessel isolation protocols commonly rely on enzymatic digestion (e.g., collagenase, trypsin, papain) which risks over-digestion, loss of mural cells, and disruption of endothelial-pericyte-astrocyte contacts.^2^^,^^3^ This protocol uses mechanical homogenization followed by dextran density-gradient centrifugation and cell-strainer purification, avoiding enzymatic incubation entirely. The approach preserves the neurovascular unit, shortens total isolation time to approximately 4 hours from perfusion to sample readiness, and yields brain vessel fragments suitable for downstream processing including immunofluorescence staining, western blot, and LC-MS/MS proteomics.

### Institutional permissions

All animal procedures were performed in accordance with the Guide for the Care and Use of Laboratory Animals and were approved by the Institutional Animal Care and Use Committee (IACUC, Protocol #20678) of the University of Southern California. Investigators performing this protocol at other institutions must obtain approval from their respective animal care committees before starting.

### Preparation of buffer A (homogenizing buffer)

**Timing: 30 min (plus 12–16 h 4°C cooling)**

6. In a 1 L beaker, add 800 mL autoclaved ddH2O and combine the dry reagents listed in the recipe for buffer A (see materials and equipment).
  a. Dissolve BSA completely on a magnetic stir plate.
  b. Add 10 mL of 100 mM Na-pyruvate in a sterile hood to keep the stock sterile.
  c. Bring the final volume to 1000 mL with autoclaved ddH2O.
7. Calibrate a pH meter with pH 7.00 buffer, then adjust buffer A to pH 7.4 dropwise with 1.0 M NaOH. Do not overshoot 7.4.

**CRITICAL:** pH drift above 7.5 compromises BSA stability and the subsequent dextran step. Always calibrate the pH meter immediately before use.

8. Transfer the buffer into a sterile hood, sterile-filter through a 0.22 μm Nalgene filter, aliquot into 50 mL tubes, and store at 4°C for up to 4 weeks.

### Preparation of buffer B (isotonic perfusion buffer)

**Timing: 30 min**

9. In a 1 L beaker, dissolve the dry reagents listed in the recipe for buffer B (see materials and equipment) in 800 mL autoclaved ddH2O.
10. Adjust pH to 7.4 with 1.0 M NaOH as for buffer A.
11. Bring to 1000 mL, sterile-filter, aliquot into 50 mL tubes, and store at 4°C for up to 4 weeks.

### Preparation of 30% dextran

**Timing: 2 h (plus autoclave and cooling time)**

12. In a sterile 250 mL bottle, add 50 mL of ddH2O warmed in a microwave for 15 s to approximately 50°C–60°C (warm to the touch but not scalding).
13. Weigh 30 g of dextran (Sigma-Aldrich, cat. no. 31390) and deliver into the bottle using a funnel.
14. Add 10 mL of 10× PBS stock solution and bring volume to 100 mL with ddH2O. Dissolve completely on a magnetic stirrer; a glass rod may be used to break up clumps.
15. Cap loosely, secure with autoclave tape, place the bottle in a water-filled beaker (water level above the dextran line), and autoclave on a 15 min liquid cycle. After the autoclave cycle finishes, tighten the cap.

**CRITICAL:** The water bath prevents dextran browning during autoclaving. Caramelized dextran has altered density and will not separate brain vessels correctly. Properly prepared dextran is clear to slightly yellow; discard if the solution appears brown or amber.

16. Cool to 20°C–25°C, then transfer to a sterile hood and aliquot in 5 mL volumes into 15 mL tubes. Store at 4°C for up to 3 months.

### Preparation of ketamine/xylazine stock

**Timing: 15 min**

17. In a sterile 15 mL tube inside a biosafety cabinet, add 1.0 mL of ketamine (100 mg/mL stock) and 0.5 mL of xylazine (20 mg/mL stock).
18. Bring to 5 mL with sterile saline. Label and store at 4°C for up to 1 week.

***Note:*** Working stock contains 20 mg/mL ketamine and 2 mg/mL xylazine. Inject 10 µL per gram body weight i.p. to deliver 200 mg/kg ketamine and 20 mg/kg xylazine, within the approved range (200–300 mg/kg ketamine / 20–30 mg/kg xylazine).

## Key resources table

**Table:** 

REAGENT or RESOURCE	SOURCE	IDENTIFIER
**Antibodies**		

Rat anti-CD31/PECAM-1 (IF 1:100)	BD Pharmingen	Cat# 550274; RRID:AB_393571
Mouse anti-PDGFR-β (IF 1:100)	Abcam	Cat# ab69506
Mouse anti-NeuN (IF 1:100)	Abcam	Cat# ab104224
Rabbit anti-Iba-1 (IF 1:100)	Biocare Medical	Cat# CP290A
Rabbit anti-PDGFR-β (WB 1:1000)	Cell Signaling Technology	Cat# 4564
Rabbit anti-PECAM-1/CD31 (WB 1:1000)	Cell Signaling Technology	Cat# 77699
Rabbit anti-β3-Tubulin (TuJ1) (WB 1:1000)	Cell Signaling Technology	Cat# 5666
Mouse anti-GFAP (IF 1:100)	Millipore Sigma	Cat# MAB3402
Rabbit anti-Claudin-5 (WB 1:1000)	Cell Signaling Technology	Cat# 49564
Rabbit anti-GAPDH (WB 1:1000)	Cell Signaling Technology	Cat# 2118
Goat anti-Rat IgG AF647 (IF 1:500)	Invitrogen	Cat# A21247
Donkey anti-Mouse IgG AF568 (IF 1:500)	Invitrogen	Cat# A10037
Donkey anti-Rabbit IgG AF568 (IF 1:500)	Invitrogen	Cat# A10042
HRP-conjugated anti-mouse IgG (WB 1:2000)	Cell Signaling Technology	Cat# 7076
HRP-conjugated anti-rabbit IgG (WB 1:2000)	Cell Signaling Technology	Cat# 7074

**Chemicals, peptides, and recombinant proteins**		

Sodium chloride (NaCl)	Sigma-Aldrich	Cat# S7653
Potassium chloride (KCl)	Sigma-Aldrich	Cat# P9541
Calcium chloride dihydrate (CaCl2·2H2O)	Sigma-Aldrich	Cat# C7902
Potassium phosphate (KH2PO4)	Sigma-Aldrich	Cat# P9791
Magnesium sulfate heptahydrate (MgSO4·7H2O)	Sigma-Aldrich	Cat# 63138
HEPES	Sigma-Aldrich	Cat# H4034
Sodium bicarbonate (NaHCO3)	Sigma-Aldrich	Cat# S6297
D-Glucose	Sigma-Aldrich	Cat# G7528
Bovine serum albumin (BSA)	Sigma-Aldrich	Cat# B4287
Sodium pyruvate, 100 mM	Gibco	Cat# 11360070
Dextran, average MW ∼70 kDa	Sigma-Aldrich	Cat# 31390
PBS powder (0.01 M)	Sigma-Aldrich	Cat# SLBL3211V
PBS solution (1×)	Gibco	Cat# 10010-023
4% paraformaldehyde in PBS	Fisher Scientific	Cat# AAJ19943K2
Normal donkey serum	Southern Biotech	Cat# 0030-01
Mounting medium with DAPI	Abcam	Cat# ab104139
RIPA buffer (10×)	Cell Signaling Technology	Cat# 9806
cOmplete Mini protease inhibitor cocktail	Roche	Cat# 11836153001
Blotting-grade blocker (nonfat dry milk)	Bio-Rad	Cat# 1706404
SignalFire ECL reagent	Cell Signaling Technology	Cat# 6883
Ketamine HCl (100 mg/mL)	MWI Veterinary Supply Co.	N/A
Xylazine HCl (20 mg/mL)	MWI Veterinary Supply Co.	N/A

**Experimental models: Organisms/strains**		

Mouse: C57BL/6J, 8-40 weeks (aged 8-10 months for CADASIL studies), both sexes	The Jackson Laboratory	Strain# 000664; RRID:IMSR_JAX:000664
Mouse: Notch3 R170C conditional knock-in (CADASIL)	Huang et al., 2026^1^	N/A

**Software and algorithms**		

ImageJ/Fiji	NIH	RRID:SCR_002285
NIS-Elements (confocal acquisition)	Nikon	RRID:SCR_014329
ImageQuant TL (western blot)	Cytiva	RRID:SCR_018374

**Other**		

Dissection microscope	Leica Microsystems	N/A
60 mm petri dish	Falcon Corning	Cat# 353002
Dumont #5 forceps	Fine Science Tools	Cat# 11251-35
Dumont #7 forceps	Fine Science Tools	Cat# 11270-20
Halsted-Mosquito hemostat	Fine Science Tools	Cat# 91309-12
Spatula-probe	Fine Science Tools	Cat# 10090-13
Iris scissors, large loops	Fine Science Tools	Cat# 14041-10
Graefe forceps	Fine Science Tools	Cat# 11051-10
Vessel cannulation forceps	Fine Science Tools	Cat# 00574-11
Safety scalpel	Fine Science Tools	Cat# 10006-12, 10010-10
Kimble Kontes Dounce homogenizer (apparatus A, 2 mL)	DWK Life Sciences	Cat# 885300-002
Low-bind 1.5 mL centrifuge tube	Eppendorf	Cat# 13-698-794
Low-bind 2.0 mL centrifuge tube	Eppendorf	Cat# 022431048
Peristaltic perfusion pump	Marshall Scientific	Cat# RD-RP1
IV butterfly needle, 27 G	Terumo	Cat# 72-5965
40 μm nylon cell strainer	Corning	Cat# 352340
100 μm nylon cell strainer	Corning	Cat# 352360
Cytospin stainless-steel slide clip	Fisher Scientific	Cat# 59-910-052
Cytospin cone with filter card	Fisher Scientific	Cat# 59-910-40
Adhesion microscope slides	VWR	Cat# 48311-703
Cytospin centrifuge (Shandon Cytospin)	Thermo Shandon	Cat# MA2763A0107
ImmEdge hydrophobic barrier pen	Vector Laboratories	Cat# H-4000
No. 1.5 cover glass	VWR	Cat# 48404-453
Confocal microscope	Nikon	A1R
Western blot imaging system	Cytiva Amersham	ImageQuant 800

## Materials and equipment

**Table:** Buffer A (homogenizing buffer)

Reagent	Final concentration	Amount (per 1 L)
NaCl	100 mM	5.844 g
KCl	4.6 mM	343.2 mg
CaCl2·2H2O	2.4 mM	353 mg
KH2PO4	1 mM	136.1 mg
MgSO4·7H2O	1 mM	246.5 mg
HEPES	15 mM	3.575 g
NaHCO3	20 mM	1.680 g
D-Glucose	10 mM	1.802 g
BSA	0.5% (w/v)	5.0 g
Na-Pyruvate (100 mM)	1 mM	10 mL
Autoclaved ddH2O	N/A	to 1 L

Adjust to pH 7.4, sterile-filter through a 0.22 μm Nalgene filter. Store at 4°C for up to 4 weeks.

**Table:** Buffer B (isotonic perfusion buffer)

Reagent	Final concentration	Amount (per 1 L)
NaCl	100 mM	5.844 g
KCl	4.6 mM	343.2 mg
CaCl2·2H2O	2.4 mM	353 mg
KH2PO4	1 mM	136.1 mg
MgSO4·7H2O	1 mM	246.5 mg
HEPES	15 mM	3.575 g
Autoclaved ddH2O	N/A	to 1 L

Adjust to pH 7.4, sterile-filter, store at 4°C for up to 4 weeks.

**Table:** 30% dextran solution

Reagent	Final concentration	Amount (per 100 mL)
Dextran	30% (w/v)	30 g
10× PBS	1×	10 mL
Autoclaved ddH2O	N/A	to 100 mL

Autoclave (liquid cycle, 15 min) with the bottle submerged in a water bath to prevent browning. Aliquot, store at 4°C for up to 3 months.

**Table:** Ketamine/xylazine working stock

Reagent	Final concentration	Amount (per 10 mL)
Ketamine (100 mg/mL stock)	20 mg/mL	1.0 mL
Xylazine (20 mg/mL stock)	2 mg/mL	0.5 mL
Sterile saline	N/A	to 5 mL

Store at 4°C, use within 1 week.

## Step-by-step method details

### Transcardial perfusion and brain collection

**Timing: 30–60 min per animal**

This section describes how to perfuse an anesthetized mouse transcardially with PBS and collect the brain into cold buffer B. Complete clearance of circulating blood is required to avoid autofluorescence in subsequent imaging and non-specific bands in western blot.

1. Anesthetize the mouse by intraperitoneal injection of ketamine/xylazine working stock (20 mg/mL / 2 mg/mL) at 10 µL per gram body weight using a 27 G × ½ in insulin needle, to deliver 200 mg/kg ketamine and 20 mg/kg xylazine.
  a. Confirm anesthesia depth by toe pinch before proceeding.

**CRITICAL:** Do not begin perfusion until all pedal reflexes are absent.

2. Place the mouse supine, open the thoracic cavity, and expose the heart.
3. Connect an IV butterfly needle to a peristaltic perfusion pump. a. Insert the needle into the left ventricle and sever the right atrium. b. Perfuse with 30–50 mL of ice-cold 0.01 M PBS-EDTA (pH 7.4) at pump setting 12.5 rpm.ricle, sever the right atrium, and perfuse with 30–50 mL of ice-cold 0.01 M PBS-EDTA (pH 7.4) at pump setting 12.5 rpm.
  a. Stabilize the heart with forceps if needed.
  b. Perfusion is complete when the liver turns pale gray.

**CRITICAL:** Incomplete perfusion leaves blood in the cerebral vessels, causing autofluorescence in confocal imaging and non-endothelial bands in western blot. See troubleshooting 1.

***Alternatives:*** A gravity-fed perfusion system or syringe pump may be used instead of a peristaltic pump. Maintain a flow rate of approximately 5–10 mL/min regardless of the system used.

4. Decapitate the animal, make a midline incision along the skull, and expose the brain.
  a. Use fine scissors and proceed with care to avoid damaging the cortex underneath.
5. Remove the whole brain with a spatula-probe and transfer it into a 60 mm petri dish containing cold buffer B on ice.

### Removal of meninges and surface vessels

**Timing: 15 min per brain**

Surface vessels and meninges must be removed before homogenization; these structures are a major source of brain vessel contamination.

6. Submerge the brain in cold buffer B.
7. Under a dissection microscope, hold the brain with vessel cannulation forceps and remove the meninges and surface vessels in a scooping motion using fine-tipped forceps.
  a. Inspect the dorsal, ventral, and lateral surfaces systematically. Pay attention to vessels at the midline and along sulci.
8. Bisect the brain along the midline with a safety scalpel. Remove the brainstem, cerebellum, and hippocampus.
  a. The two cortical hemispheres are retained for white matter removal.

### White-matter removal

**Timing: 30 min for two hemispheres**

Residual white matter overlays the brain vessel layer after density-gradient separation and generates sheet-like contamination that obscures immunofluorescence signal. Thorough white matter removal is therefore essential.

9. Keep each hemisphere in cold buffer B in a chilled petri dish.
10. Using Graefe forceps, invert the hemisphere to expose the white matter. Gently peel and scrape the white matter tract from the medial cortical surface.

**CRITICAL:** Do not discard cortical gray matter. If unsure, rinse the hemisphere in buffer B and re-inspect: residual white matter is denser and appears glossy compared to gray matter. See troubleshooting 2.

11. Transfer the cleaned hemispheres to a fresh petri dish containing cold buffer A. Cut into small (∼2 mm), uniform flakes using a sterile scalpel to facilitate homogenization.

### Brain tissue homogenization

**Timing: 15 min**

This step shears cortical tissue into a uniform cloudy suspension while preserving brain vessel integrity. Over-homogenization damages vessels; under-homogenization reduces yield.

12. Pre-coat a 2 mL Kimble Kontes Dounce homogenizer (apparatus A) with 1 mL buffer A. Mark the 1 mL fill line with a permanent marker. Pump the pestle up and down several times to coat both pestle and tube, then discard the buffer.

***Alternatives:*** A glass Dounce homogenizer is recommended for optimal vessel preservation. Plastic disposable homogenizers may be used but can generate more vessel fragmentation due to less precise pestle clearance.

13. Transfer the cortical tissue into the Dounce tube using a buffer A-coated pipette tip. Do not exceed the 1 mL fill line.
14. Homogenize with 20–30 slow strokes on ice until the suspension is uniform and cloudy.

**CRITICAL:** Use slow, steady strokes. Aggressive homogenization fragments vessels and reduces yield. See troubleshooting 4.

### Capillary-depleted parenchyma and brain vessel separation

**Timing: 45 min**

Dextran density-gradient centrifugation separates the capillary-depleted brain parenchyma (floating supernatant) from the brain vessel fraction (bottom pellet).

15. Transfer 1 mL of 30% dextran into a pre-chilled 2.0 mL low-bind tube.

**CRITICAL:** Dextran with an average molecular weight of ∼70 kDa (Sigma-Aldrich, cat. no. 31390) is required; other molecular weight dextrans will not produce the correct density for vessel separation.

16. Add the homogenate from the previous step to the same tube. Cap tightly and vortex thoroughly (10 s) to mix.
17. Centrifuge at 6,000 × *g* for 20 min at 4°C.
18. Using a wide-bore 1 mL pipette tip (snipped off with sterile scissors), carefully collect the opaque top layer (capillary-depleted parenchyma; approximately 0.5mL) into a labeled low-bind 1.5 mL tube for downstream analyses. a. Add 1 mL cold buffer B to the capillary-depleted brain, mix gently, and centrifuge at 300 × *g* for 10 min at 4°C. b. Aspirate the supernatant and resuspend the pellet in buffer B. Repeat the wash a total of 3 times to remove residual glucose and dextran.
19. In the original 2.0 mL tube, discard the middle dextran layer carefully without disturbing the brain vessel pellet.

**CRITICAL:** Do not aspirate into the pellet. Residual dextran inhibits downstream lysis and immunostaining. See troubleshooting 5.

20. Resuspend the brain vessel pellet in 1 mL cold buffer A using a buffer A-coated pipette tip. Pipet up and down on ice until the suspension is uniform.

### Filtration to remove gross debris

**Timing: 10 min**

A single pass through a 100 μm nylon strainer removes large tissue clumps and residual debris while retaining the full brain vessel preparation, including capillaries, arterioles, and venules.

21. Place a 100 μm nylon cell strainer on top of a 50 mL conical tube. Pre-wet with 1 mL cold buffer A.
22. Pour the resuspended brain vessel pellet through the 100 μm strainer. Collect the filtrate.
23. Rinse the strainer with 1–2 mL cold buffer A to recover brain vessel fragments retained on the mesh. Pool the rinse with the filtrate in the 50 mL conical tube; this combined fraction contains the complete brain vessel preparation. Transfer the combined filtrate to a pre-chilled 15 mL conical tube.
24. Centrifuge the combined filtrate at 300 × *g* for 10 min at 4°C. Aspirate the supernatant carefully without disturbing the pellet. Resuspend the brain vessel pellet in 0.5–1.0 mL cold buffer A.

**Pause point:** The brain vessel pellet can be held on ice for up to 2 h before downstream processing. For proteomics, snap-freeze the pellet in liquid nitrogen and store at −80°C. Samples can be shipped on dry ice to the proteomics core at this stage.

***Note:*** A single brain vessel preparation from two cortical hemispheres of one adult mouse typically yields 30–80 μg total protein. Western blot requires approximately 20 μg per lane; LC-MS/MS proteomics requires 30–35 μg. At typical yields, one preparation can be split for both western blot and proteomics. For combined proteomics and phosphoproteomics, scale up to ≥4 animals per group and pool 100 μg per sample for TiO2/IMAC Fe-NTA or other phosphopeptide enrichment assays prior to LC-MS/MS. See Huang et al.^1^ for the specific mass spectrometer, LC system, and acquisition parameters used in the CADASIL study.

### Immunofluorescence staining of isolated brain vessels

**Timing: 18 h, including 12–16 h primary antibody incubation**

Cytospin of the brain vessel suspension onto adhesion slides allows direct immunofluorescence staining to assess morphology and purity with cell-type-specific markers.

25. Load 20 μL of brain vessel suspension into a cytospin cone seated on an adhesion slide in a stainless-steel slide clip. Balance with a second loaded clip on the opposite side of the rotor.
  a. Seat the filter card on the slide clip, then position the cytospin cone on top of the filter card with the narrow end pointing toward the slide.
  b. Pipet the vessel suspension into the well of the cone. Avoid introducing air bubbles.
  c. Place the assembled clip into the rotor, ensuring the weighted side faces outward cytospin cone seated on an adhesion slide in a stainless-steel slide clip. Balance with a second loaded clip on the opposite side of the rotor.
26. Cytospin at 1,500 × rpm for 5 min.

***Alternatives:*** If a cytospin centrifuge is unavailable, coat adhesion slides with poly-L-lysine, pipet 20 μL of vessel suspension onto the coated area, and allow to adhere for 10 min at 20°C–25°C before fixing.

27. Carefully remove the slide, taking care not to disturb the adhered brain vessels. Draw a hydrophobic barrier around the vessel spot with an ImmEdge pen.
28. In a fume hood, fix with 50 μL of 4% PFA in PBS at 20°C–25°C for 10 min. Wash 3× with PBS.
29. Block with 5% donkey serum in PBS for 30 min at 20°C–25°C.
30. Incubate with primary antibodies diluted 1:100 in blocking buffer 12–16 h at 4°C in a humidified chamber. Suggested panel: anti-CD31 (endothelial), anti-PDGFR-β (pericytes), anti-Iba-1 (microglia, purity control), anti-NeuN (neurons, purity control).
31. Wash 3 × 5 min with PBS.
32. Incubate with species-matched AlexaFluor-conjugated secondary antibodies diluted 1:500 in blocking buffer for 2–3 h at 20°C–25°C in the dark.
33. Wash 3 × 5 min with PBS.
34. Mount with DAPI Fluoromount-G and image on a confocal microscope (Figure 2).

**Figure 1:**
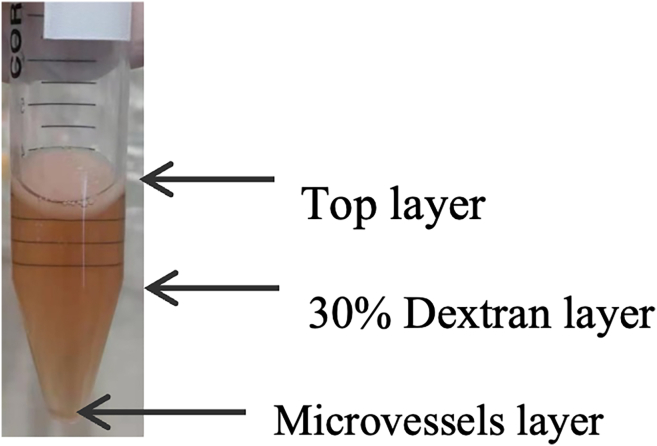
Fractionation of mouse cortical homogenate after dextran density-gradient centrifugation Representative image of a 1.5 mL tube after 6,000 × *g*, 20 min, 4°C centrifugation showing the cloudy parenchymal top layer, clear middle dextran layer, and white brain vessel pellet at the bottom.

**Figure 2:**
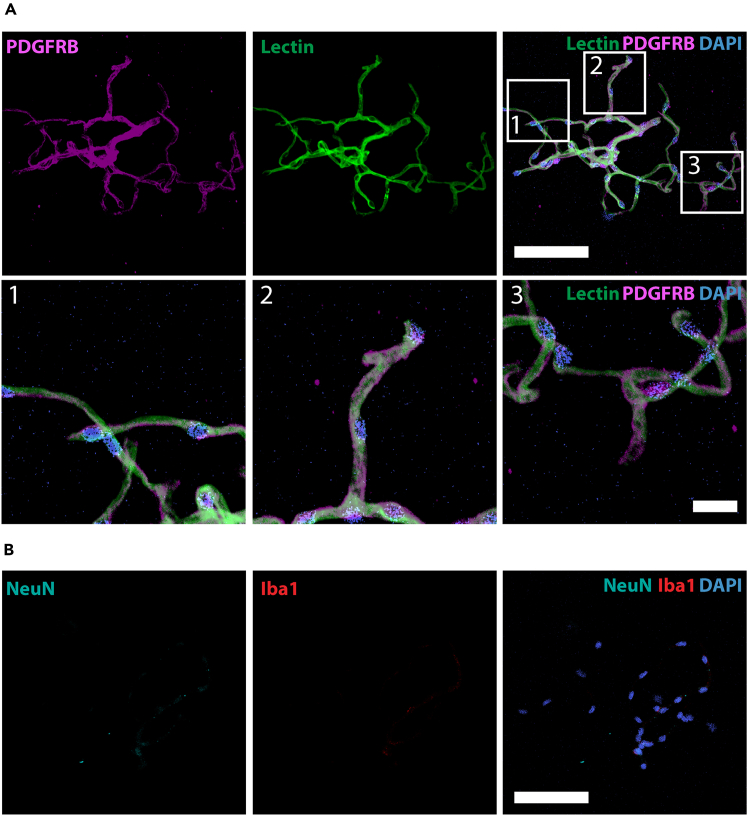
Immunofluorescence characterization of isolated brain vessels (A) Cytospun brain vessels stained with lectin (endothelial cells) and antibodies against PDGFR-β (pericytes). (B) NeuN (neurons, purity control), and Iba-1 (microglia, purity control); nuclei counterstained with DAPI. Scale bar: 50 µm. Reprinted and adapted with permission from Huang et al.^1^

***Note:*** Negative controls: omit each primary antibody in one parallel slide to assess secondary antibody specificity. See troubleshooting 3.

### Purity assessment by western blot

**Timing: 24 h, including 12–16 h primary antibody incubation**

Western blot against cell-type-specific markers provides a quantitative complementary readout of brain vessel enrichment over the capillary-depleted parenchyma fraction.

**Figure 3:**
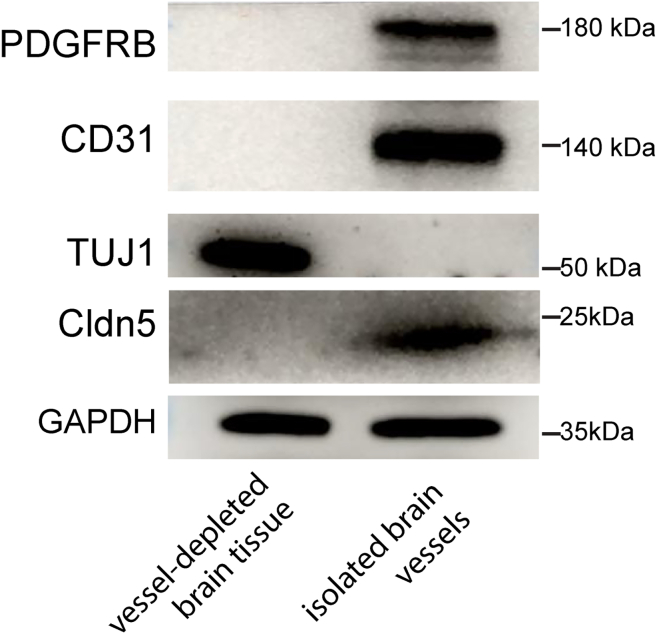
Western blot of isolated brain vessel and capillary-depleted parenchyma fractions Membranes were probed with antibodies against PDGFR-β, CD31, TuJ1 (neurons), and Claudin-5 (endothelial tight junctions). GAPDH serves as a loading reference. Reprinted and adapted with permission from Huang et al.^1^

35. Pellet the remaining brain vessel suspension at 300 × g for 10 min at 4°C. Discard supernatant.
36. Add 80 μL of 1× RIPA buffer supplemented with protease inhibitor cocktail. Resuspend by pipetting.
37. Sonicate on ice: 20 kHz, 5 s on/10 s off, 3 cycles.
38. Clear the lysate at 12,000 × *g* for 15 min at 4°C. Transfer the supernatant to a fresh low-bind 1.5 mL tube.
39. Determine protein concentration with a BCA assay.
40. Mix equal amounts of protein (typically 10**–**20 μg) with SDS loading buffer, denature at 95°C**–**100°C for 5**–**10 min.
41. Resolve on a 10% SDS-PAGE gel: 80 V through stacking gel (20**–**30 min), then 120 V through resolving gel (60**–**90 min).
42. Transfer to PVDF membrane by wet transfer in Towbin buffer at 100 V for 60 min at 4°C.
43. Block with 5% nonfat dry milk in TBS-T for 1 h at 20°C–25°C.
44. Cut the membrane between molecular weight markers if multiple antibodies will be used. Incubate 12–16 h at 4°C with primary antibodies (1:1000) against PDGFR-β, CD31, TuJ1, Claudin-5, and GAPDH.
45. Wash 3 × 5 min with TBS-T. Incubate with HRP-conjugated secondary antibodies (1:2000) for 2–3 h at 20°C–25°C.
46. Wash 3 × 5 min with TBS-T. Develop with SignalFire ECL reagent and image (Amersham ImageQuant 800; Figure 3). See troubleshooting 6.

### Sample preparation for LC-MS/MS proteomics

**Timing: 2 h (plus 12–16 h shipment on dry ice if required)**

This section describes preparation of the brain vessel pellet for quantitative liquid chromatography-tandem mass spectrometry (LC-MS/MS) proteomic and phosphoproteomic profiling as described in Huang et al.^1^

***Note:*** For downstream LC-MS/MS applications, use proteomics-grade or LC-MS-grade chemicals where available (e.g., TEAB, SDS, sequencing-grade trypsin) to minimize contaminants that interfere with mass spectrometry.

47. If the brain vessel pellet was stored at −80°C, thaw on ice. If proceeding directly from Step 24, use the freshly pelleted material.
48. If not already snap-frozen in Step 24, snap-freeze the pellet in liquid nitrogen and store at −80°C until lysis. Samples can be shipped on dry ice to the proteomics core.in liquid nitrogen and store at −80°C until lysis.

**CRITICAL:** Do not lyse in RIPA or other SDS-free detergents if the downstream application is LC-MS/MS. The lysis buffer must be compatible with trypsin digestion and mass spectrometry.

49. Dissolve each pellet in 500 μL ice-cold lysis buffer (0.5 M triethylammonium bicarbonate (TEAB), 0.05% sodium dodecyl sulfate (SDS)).
50. Lyse by tip sonication on ice with a Q700 sonicator (QSonica) at amplitude 10, 2 s on/2 s off pulses, 20 s total processing time per sample.
51. Centrifuge the lysate at 20,000 × *g* for 10 min at 4°C. Transfer the supernatant to a fresh low-bind tube.
52. Determine protein concentration using the Qubit Protein Assay Kit (Thermo Fisher Scientific, Q33211) on a Qubit 4 fluorometer. Typical yield from two cortical hemispheres of one adult mouse is 30–80 μg of protein in 500 μL lysate.
53. Proceed with reduction, alkylation, tryptic digestion, TMT labeling, and LC-MS/MS acquisition following standard proteomics core workflows. Reduce with 5 mM tris(2-carboxyethyl)phosphine (TCEP) at 37°C for 1 h. Alkylate with 10 mM iodoacetamide (IAA) at 20–25°C for 30 min in the dark. Digest with sequencing-grade trypsin (1:50 enzyme:protein) 12–16 h at 37°C. Label peptides with TMT reagents per manufacturer instructions. Acquire LC-MS/MS data on an Orbitrap mass spectrometer coupled to an EASY-nLC system.cquisition following standard proteomics core workflows. See Huang et al.^1^ for the specific mass spectrometer, LC system, and acquisition parameters used in the CADASIL study.

***Note:*** For combined proteomics and phosphoproteomics, scale up to ≥4 animals per group and pool 100 μg per sample for TiO2/IMAC Fe-NTA or other phosphopeptide enrichment assays prior to LC-MS/MS.

## Expected outcomes

Following dextran density-gradient centrifugation, three fractions are visible in the 2.0 mL tube: a cloudy top layer containing capillary-depleted parenchyma, a clear middle dextran layer, and a white brain vessel pellet (Figure 1). Yield from two cortical hemispheres of one adult C57BL/6J mouse is typically 400–600 μL of brain vessel suspension suitable for cytospin and protein analysis.^1^^,^^4^

Immunofluorescence of cytospun brain vessels shows strong staining for CD31 and PDGFR-β, outlining intact endothelial-pericyte interactions, with minimal signal for NeuN or Iba-1 (Figure 2). The absence of neuronal and microglial markers confirms removal of parenchymal contaminants. Some GFAP-positive astrocytic end-feet may remain associated with the brain vessel surface, consistent with preservation of the neurovascular unit. Adapted with permission from Huang et al.^1^

Western blot of the brain vessel lysate shows enrichment of CD31, PDGFR-β, and Claudin-5 relative to the capillary-depleted parenchyma fraction, with the neuronal marker TuJ1 detectable only in the parenchymal fraction (Figure 3). GAPDH serves as a loading control. Adapted with permission from Huang et al.^1^

## Limitations

This protocol relies on mechanical homogenization, which preserves the neurovascular unit but produces variable brain vessel fragment sizes between operators. Strict adherence to pestle stroke count and stroke speed is required for reproducibility. Residual white matter contamination can occur if removal is incomplete, leading to sheet-like background in immunofluorescence. After dextran centrifugation, minor cross-contamination from the parenchymal top layer can reach the brain vessel pellet if aspiration is not careful. The protocol is optimized for adult mouse cortex; application to other brain regions, neonatal mice, or human post-mortem tissue may require adjustment of homogenization vigor or dextran concentration. The final preparation is a mixed vessel population (capillaries, arterioles, venules together with associated mural cells and some astrocytic end-feet), which is an advantage for bulk proteomics but limits cell-type-specific resolution. For cell-type-resolved analyses, downstream FACS sorting or single-cell or single-nucleus RNA sequencing is recommended, as detailed in prior studies^1^^,^^5^^,^^6^ with existing protocols.^7^^,^^8^

## Troubleshooting

### Problem 1 (related to steps 1–5: Transcardial perfusion and brain collection)

The final brain vessel suspension shows red or brown tinge, and confocal imaging shows high autofluorescence in the red channel.

### Potential solution

• Extend perfusion volume to 50–60 mL and verify liver blanching before stopping.
• Confirm the butterfly needle is correctly placed in the left ventricle (not the right ventricle) and that the right atrium is fully severed.
• Check pump flow rate (pump setting 12.5 rpm) and tubing for air bubbles that interrupt flow.

### Problem 2 (related to steps 9–11: White-matter removal)

Immunofluorescence images show sheet-like tissue covering the brain vessels. Under immunofluorescence, incomplete myelin removal appears as dense, filamentous background that obscures CD31 and PDGFR-β signal, particularly in the deeper focal planes. Myelin debris stains positively for MBP (myelin basic protein) and can be distinguished from vessel structures by its sheet-like rather than tubular morphology.

### Potential solution

• After removing meninges and deep structures, place the hemisphere in a clean dish with a shallow buffer B pool. Press the cortex gently against the dish bottom on ice and scrape residual white matter with a safety scalpel.
• Transfer the hemisphere into fresh buffer B between scraping passes; residual white matter fragments detach into the buffer and become easier to identify.
• Inspect under a dissection microscope before homogenization; white matter appears as glossy strands.

### Problem 3 (related to steps 25–34: Immunofluorescence staining and steps 35–46: Purity assessment by western blot)

NeuN or Iba-1 signal is detected in the brain vessel cytospin, and TuJ1 bands appear in the western blot of the brain vessel fraction.

### Potential solution

• Discard the entire top parenchymal layer with a wide-bore tip before accessing the dextran layer. Change tips when moving to the middle dextran layer.
• Use a fresh tip again when collecting the bottom brain vessel pellet and avoid contacting the tube wall where residual parenchymal tissue may stick.
• If filtration was used, repeat the 100 μm strainer wash step with an additional 20–30 mL of cold buffer A. Otherwise, add an extra buffer-A wash and re-centrifuge at 300 × g for 10 min at 4°C before re-suspending.

### Problem 4 (related to steps 12–14: Brain tissue homogenization)

Very low brain vessel yield in the final pellet; the strainer (if used) appears nearly empty, or the resuspension is clear rather than cloudy.

### Potential solution

• Reduce the number and force of pestle strokes; overstrong homogenization shears vessels into fragments too small to recover.
• Ensure the homogenizer and all buffers are pre-chilled; warm temperatures accelerate tissue disintegration.
• Verify that the dextran concentration is 30% (w/v) and has not been diluted by residual water during autoclaving.

### Problem 5 (related to steps 15–20: Capillary-depleted parenchyma and brain vessel separation)

The three-layer separation after centrifugation is not distinct, or the brain vessel pellet is diffuse.

### Potential solution

• Re-check centrifuge speed (6,000 × *g*) and time (20 min). Lower speeds do not separate the fractions.
• Vortexing was insufficient to thoroughly mix dextran and homogenate. Re-vortex sample for 30 sec, and perform centrifuge step again.
• Use freshly made dextran; dextran older than 3 months can lose density.
• Ensure the centrifuge rotor is balanced with a matched tube of the same volume.

### Problem 6 (related to steps 35–46: Purity assessment by western blot)

GAPDH signal is much stronger in the parenchyma fraction than in the brain vessel fraction, confounding normalization.

### Potential solution

• Normalize to total protein by stain-free or Ponceau S staining instead of a single housekeeping protein.
• Include a vascular-enriched reference such as β-actin or Claudin-5 in the normalization strategy.

## Resource availability

### Lead contact

Further information and requests for resources and reagents should be directed to and will be fulfilled by the lead contacts, Ruslan Rust (rrust@usc.edu) and Marcelo P. Coba (coba@usc.edu).

### Technical contact

Technical questions on executing this protocol should be directed to and will be answered by the technical contact, Veronica Clementel (vac_924@usc.edu).

### Materials availability

This study did not generate new unique reagents or materials.

### Data and code availability

This protocol did not generate new datasets or code. Representative western blot images and immunofluorescence images shown in Figures 2 and 3 have been adapted from Huang et al.^1^ with permission.

## Acknowledgments

This work is supported by funding from the Swiss 3R Competence Center (OC2020-002), the 10.13039/501100001711Swiss National Science Foundation (PZ00P3_216225), USC Dean’s Pilot Funding Award (000092), and the Epstein Family Breakthrough Alzheimer’s Research Award (000153). The graphical abstract was generated in BioRender and PowerPoint.

## Author contributions

Conceptualization, M.P.C. and R.R.; methodology, Y.H., V.C., and C.T.-S.; investigation, Y.H., V.C., M.Z., Kate Martinez, Karen Martinez, G.S., and C.T.-S.; LC-MS sample preparation and proteomics analysis, V.C., M.P.C., and R.R.; supervision, K.K., M.P.C., and R.R.; writing – original draft, Y.H., M.P.C., and R.R.; writing – review and editing, all authors.

## Declaration of interests

The authors declare no competing interests.

## Declaration of generative AI and AI-assisted technologies in the writing process

During the preparation of this work, the authors used Claude (Claude Opus 4.8; Anthropic) in order to improve the language and readability of the manuscript. After using this tool, the authors reviewed and edited the content as needed and take full responsibility for the content of the publication.
